# Retraction: Immunosuppressant MPA Modulates Tight Junction through Epigenetic Activation of MLCK/MLC-2 Pathway via p38MAPK

**DOI:** 10.3389/fphys.2017.00724

**Published:** 2017-09-19

**Authors:** Frontiers Editorial Office

The Journal and Chief Editors retract the 22 December 2015 article cited above. Based on information discovered after publication and reported to Frontiers in January 2017, the article was examined, revealing image duplications in Figures 1B, 2E.

As this duplication breaches Frontiers guidelines and could not be sufficiently explained by the authors, the article has been retracted. The retraction of the article was approved by the Field Chief Editor for Frontiers in Physiology. The authors do not agree to the retraction and the notice.

